# Texture-induced vibrations in the forearm during tactile exploration

**DOI:** 10.3389/fnbeh.2012.00037

**Published:** 2012-07-06

**Authors:** Benoit Delhaye, Vincent Hayward, Philippe Lefèvre, Jean-Louis Thonnard

**Affiliations:** ^1^Institute of Neuroscience, Université Catholique de LouvainBrussels, Belgium; ^2^Institute of Information and Communication Technologies, Electronics and Applied Mathematics, Université Catholique de LouvainLouvain-la-Neuve, Belgium; ^3^Institut des Systèmes Intelligents et de Robotique, Université Pierre-et-Marie CurieParis, France

**Keywords:** texture, roughness, vibration, touch

## Abstract

Humans can detect and discriminate between fine variations of surface roughness using active touch. It is hitherto believed that roughness perception is mediated mostly by cutaneous and subcutaneous afferents located in the fingertips. However, recent findings have shown that following abolishment of cutaneous afferences resulting from trauma or pharmacological intervention, the ability of subjects to discriminate between textures roughness was not significantly altered. These findings suggest that the somatosensory system is able to collect textural information from other sources than fingertip afference. It follows that signals resulting of the interaction of a finger with a rough surface must be transmitted to stimulate receptor populations in regions far away from the contact. This transmission was characterized by measuring in the wrist vibrations originating at the fingertip and thus propagating through the finger, the hand and the wrist during active exploration of textured surfaces. The spectral analysis of the vibrations taking place in the forearm tissues revealed regularities that were correlated with the scanned surface and the speed of exploration. In the case of periodic textures, the vibration signal contained a fundamental frequency component corresponding to the finger velocity divided by the spatial period of the stimulus. This regularity was found for a wide range of textural length scales and scanning velocities. For non-periodic textures, the spectrum of the vibration did not contain obvious features that would enable discrimination between the different stimuli. However, for both periodic and non-periodic stimuli, the intensity of the vibrations could be related to the microgeometry of the scanned surfaces.

## Introduction

Humans have the ability to detect fine features of textured surfaces and to discriminate between them using *direct, active finger touch* (Katz, 1925/1989; Sathian et al., 1989; Connor et al., 1990; Libouton et al., 2010). During this exploration mode, the tactile roughness discrimination is believed to be mediated through two combined mechanisms. Firstly, a spatial code represents texture information spatially, through the distributed activation of populations of adjacent mechanoreceptors. This channel is thought to rely on slowly adapting (SA1) mechanoreceptors (Connor et al., 1990 and Connor and Johnson, 1992) and the density of the mechanoreceptors naturally limits its resolution. Secondly, a temporal code represents the time-dependent variations of the finger-surface interaction due to their relative movement. Depending on the temporal frequency of the stimulation, either rapidly adapting (RA) or Pacinian (PC) systems are thought to encode these vibrations (Muniak et al., 2007). Textured surfaces can also be felt *indirectly*, namely by actively exploring a surface through a probe being held in the hand (indirect touch) (Klatzky and Lederman, 1999; Yoshioka et al., 2007). During this second scanning mode, roughness is necessarily encoded through vibrations transmitted through the probe to receptors in the hand.

Yoshioka et al. (2007) compared the roughness discrimination during direct and indirect touch exploration of various textured surfaces encountered in daily life (e.g., corduroy, paper, and rubber). The authors found that (1) the roughness discrimination was similar in both scanning modes and (2) the perceived roughness was correlated with the vibratory power measured on the probe. In a more recent study the same group reported that the roughness constancy was preserved in both direct and indirect touch. However, this constancy was not preserved during passive scanning, indicating the importance of hand movement and proprioception (Yoshioka et al., 2011).

Recent findings in direct active finger touch (Libouton et al., 2012) have shown that the tactile roughness discrimination performance was unaffected by entrapment or traumatic section of the median nerve at the wrist. They confirmed this finding in healthy subjects who were given an anesthetic ring bloc and were still capable to perform a tactile roughness discrimination task. The authors concluded that if the innervation of the finger pad was compromised, information about textures could be captured and encoded by remote mechanoreceptors located in more proximal tissues, where the innervation was intact. In this case, they suggested that the finger might act like a probe, transmitting the vibrations to remote receptors.

The Pacinian afferents are the primary receptors encoding these cutaneous vibrations (Hollins and Bensmaïa, 2007; Johansson and Flanagan, 2009). They are present in the subcutaneous layer of the skin but are also found near tendons, periarticular and interosseus ligaments and muscles (Mountcastle, 2005). The discovery of Hunt (1961) highlighted the existence of very sensitive RA vibration receptors being situated in the interosseus nerve of the hind limb of cats. These receptors that respond to vibrations transmitted through the footpad act “almost like a seismograph.” Hunt (1961) characterized them as Pacinian corpuscles having a very high sensitivity. As a consequence, very small vibrations transmitted through the skin and soft tissues, even applied at a considerable distance, readily evoked vigorous discharges of these Pacinian corpuscles.

The findings of Libouton et al. (2012) may be viewed as an instance of perceptual constancy, that is, subjects with de-afferented extremities tended to maintain a stable perception of roughness in spite of profound changes in the conditions under which it was acquired. Roughness constancy is a remarkably robust phenomenon, it is therefore more informative to identify when constancy breaks down rather than when it occurs. The invariant quantity that is perceived can be represented by a function *s*(*x,y*) that represents the profile of surface. The perceptual task faced by the brain is to acquire specific attributes of *s* though a complex transduction process involving tribology, contact mechanics and other physics. For simplicity, and without loss of generality to the foregoing argument, let us consider one dimension only. A contact, then, is represented by an interval, $\bar{x}$, that represents the region of contact (which can vary from 1 cm for a bare finger to a few μm for a sharp probe). During scanning the felt signal is driven by $s\left(\bar{x}\left(t\right)\right)$ through the physics of the probe-surface interaction. From the chain rule, $s\left(\bar{x}\left(t\right)\right)$ has a temporal gradient of the form, $s\prime \left(\bar{x}\right)d\bar{x}/dt=s\prime \left(\bar{x}\right)\bar{v}\left(t\right)$, that is, the product of the surface gradient filtered through the surface contact with the relative velocity of the contact region. This expression, *ceteris paribus*, makes the ambiguous character of the stimulation evident; shifting the spatial spectrum of a surface has the same effect as changing the scanning velocity. The surface spatial gradient *per se*, *s′* (x)—available through direct finger contact but not through a probe—varies through time when there is relative movement. It may or it may not participate in the perception of specific attributes of the surface. It is known, however, that the brain can estimate $\bar{v}$ from this quantity (Essick et al., 1988). To further clarify the quantities involve, let us call *v* the surface scanning velocity optained through limb movement to distinguish it from $\bar{v}$ acquired though skin afference. Using these definitions, Table 1 summarizes qualitatively previous behavioral results regarding roughness constancy. All these results are uniformly explained by the brain's ability to learn and maintain time-free representations of surfaces of the form *s(x)*, termed *spatiotopic*, accessed through the temporal gradients, $s\prime \left(\bar{x}\right)v\left(t\right)$ or $s\prime \left(\bar{x}\right)\bar{v}\left(t\right)$, if and only if the scanning velocity is available (cases 6 and 8 in Table 1). This observation, when related to the findings of Libouton et al. (2012), motivates the present study aimed at determining how subjects with de-afferented extremities could have access to $s\prime \left(\bar{x}\right)v\left(t\right)$.

**Table 1:** **Tabulation of previous results relating to texture constancy which are all explained by the brain's ability to access specific attributes of the invariant surface descriptor *s*(*x*) through the temporal gradient $s\prime \left(\bar{x}\right)v\left(t\right)$**.

**Study and main condition**	**Task**	**Movement generation**		**Quantities available**				**Constancy**	
				***s′*(*x*(*t*))**	**$s\left(\bar{x}\right)\left(t\right)$**	***v*(*t*)**	**$\bar{v}\left(t\right)$**		**Case**
Katz (1925/1989), natural surfaces. Lederman (1981) gratings of constant periodicity; bare finger.	Roughness estimation	Self		yes	yes	yes	yes	yes	(1)
		External		yes	yes	no	yes		
Lamb (1983), Meftah et al. (2000), raised dots constant periodicity, bare finger.	Spatial period discrimination Roughness estimation	Self		yes	yes	yes	yes	yes	(2)
		External		yes	yes	no	yes		
Hughes et al. (2007), raised dots with spatial period *gradient*, bare finger.	Spatial period *gradient* discrimination	Self		yes	yes	yes	yes	yes	(3)
Lawrence et al. (2007), gratings of constant periodicity; bare finger, rigid probe.	Roughness estimation	Self	Finger	yes	yes	yes	yes	yes	(4)
			Probe	yes	no	yes	no		
Wiertlewski et al. (2011a), natural surfaces, bare finger via causality inversion.	Identification and spatial period discrimination	Self		yes	no	yes	no	yes	(5)
		External		yes	no	no	no	*no*	(6)
Yoshioka et al. (2011) natural surfaces; bare finger, rigid probe	Roughness estimation	Self	Finger	yes	yes	yes	yes		
			Probe	yes	no	yes	no	yes	(7)
		External	Finger	yes	yes	no	yes		
			Probe	yes	no	no	no	*no*	(8)
Libouton et al. (2012) natural surfaces; de-afferented extremities	Roughness estimation	Self		yes	no	yes	no	yes	(9)

If v(t) is inaccessible, constancy breaks down (cases 6, 8).

Taken together these findings suggest that the somatosensory system, provided that sufficient information is available, is able to collect textural information from other sources than fingertip afferences. Vibrations generated during the scanning of textured surfaces were hypothesized to propagate through the finger and the hand, and stimulate receptors populations in regions far away from the contact region. If present in regions that are not affected by the subject trauma or by pharmacological intervention, these vibrations may explain the ability to perceive the main features of textures scanned with a finger, that is, without the benefits of the skin in contact with the surface. Moreover, there is nothing against the possibility of healthy subject to take advantage of this information too.

This hypothesis was tested in the present study by recording the vibrations propagating in the finger, hand, and wrist of subjects during active exploration of textured surfaces with the fingertip. In addition, the spectrum and the magnitude of the measured vibrations taking place in the forearm tissues were analyzed in order to test whether they were correlated to the characteristics of the explored texture surfaces.

## Materials and methods

### Subjects

Six healthy volunteers participated in the study (four males and two females, ranging in age from 25 to 30 years, five over the six subjects were right-handed). They were asked to carefully wash their right hand 10 min before the experiment. All the subjects gave their informed consent and the local ethical committee approved the experimental protocol.

### Stimuli

Periodic and non-periodic rough surfaces were used in the present study for a total of eight different textures. Five periodic stimuli with known spectral characteristics and three sandpapers with ISO scaling of the different roughness levels were compared.

**Figure 1:**
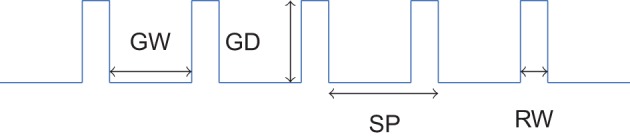
**Profile of periodic square wave gratings**. Values of SP, RW, GW, and GD are shown in Table 2.

The five different grooved surfaces were made of polyurethane resin. The grating was of periodic square waveform. The spatial period of the waves was chosen between 0.16 mm and 1.6 mm. It is assumed that textures with spatial period under 200 μm need relative movement to be felt (“duplex theory,” Hollins and Risner, 2000 and Hollins and Bensmaïa, 2007). With the chosen range of spatial period, we covered thus the two side of this theory. The profile of the wave form is shown on Figure 1. All five samples had the same dimension ratio. Following the classical studies (Taylor and Lederman, 1975), the groove width was three times the ridge width, and was equal to the groove depth. Table 2 summarizes the geometrical dimensions of the five periodic stimuli.

**Table 2:** **Set of periodic stimuli used for the experiment: geometrical dimensions of the grooved surfaces made of polyurethane resin**.

	**Spatial period (SP = λ) (μm)**	**Ridge Width (RW) (μm)**	**Groove Width (GW) (μm)**	**Groove Depth (GD) (μm)**
P1	160	40	120	120
P2	240	60	180	180
P3	400	100	300	300
P4	800	200	600	600
P5	1600	400	1200	1200

Three different sandpaper grit sizes (P80, P240, P800) were used with P80 being the coarser and P800 the thinner (see Table 3).

**Table 3:** **Set of sandpapers used for the experiment: geometrical properties**.

**ISO/FEPA grit designation (μm)**	**Average particle diameter**	**Roughness**
P800	22	Very fine
P240	58	Medium
P80	201	Coarse

### Apparatus

**Figure 2:**
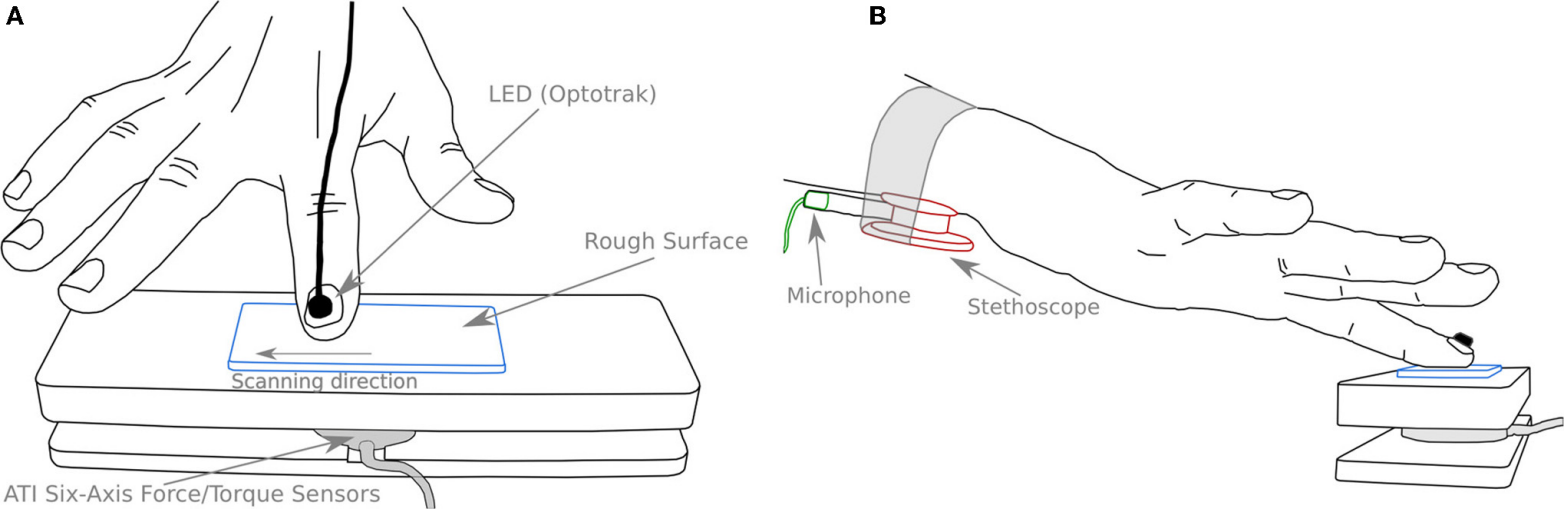
**Schematic view of the experimental setup. (A)** The stimulus (blue) is fixed on the upper part of the apparatus. A force/torque sensor (gray) measures normal and tangential forces. A LED (thick black) is glued to the subject's finger to measure its position. **(B)** A stethoscope (red) is securely fastened to the wrist of the subject. A microphone (green) is inserted in the manifold of the stethoscope and records pressure variations in it. The scanning direction was always from left to right.

A force platform was designed in order to securely fix the different stimuli and measure the contact forces (normal and tangential to the surface). A schematic view of the apparatus is shown on Figure 2. A forces and torques transducer (Mini40 F/T transducer, ATI Industrial Automation, NC, USA) was placed between the table and the support in order to measure the normal and tangential force (NF and TF, respectively) applied by the finger during scanning. The stimuli were fixed in the center of the apparatus (see Figure 2A). The position of the subject's finger was measured by means of an optical tracking system with LED markers bonded to the nail (Optotrak, Northern Digital Inc., Waterloo, Ontario, Canada). The vibrations created by the scanning of the rough surfaces were recorded using a stethoscope (Classic II S.E., 3 M Littmann, Neuss, Germany) with the bell side fastened under the flexor tendons of the wrist, where the best signal could be recorded (see Figure 2B). The pressure variations caused by the normal displacement of the skin under the stethoscope bell was sensed by a high sensitivity microphone (4060 Omnidirectional Hi-Sens, DPA Microphones, Alleroed, Denmark) inserted in the manifold of the stethoscope (see Figure 2B). A computer display was placed in front of the subject in order to give him/her a feedback of the NF applied on the rough surface and of the velocity of the finger during the scanning.

### Experimental procedure

The subject was sitting comfortably in front of the apparatus. He/she was first instructed to perform several scans, from left to right on the textured surface using the right index finger. During these strokes, he/she was asked to maintain the NF and the scanning velocity constant and as close as possible to an instructed value.

Three different NF (1, 2, and 4 N) and three different exploration velocities (50, 100, and 150 mm/s) were instructed (nine different conditions). The forces and velocities were chosen in order to correspond to values adopted naturally during tactile exploration (see Gamzu and Ahissar, 2001; Smith et al., 2002; Libouton et al., 2010; Skedung et al., 2011). Given these scanning velocities, we expected to find the fundamental frequencies for periodic stimuli ranging from 30 Hz to 1 kHz (*f* = *v*/λ). For the sandpapers, the NF was restricted to 1 N because higher forces caused skin irritation. In each condition, a block of acquisition was triggered for 15 sec once the subject was trained thereby capturing from 3 to 10 scanning movements depending on the scanning velocity.

### Data acquisition

The acquisition was realized using a custom made Labview software. The forces and sound signals were acquired with a digital-to-analog, analog-to-digital data acquisition system (DAQ 6071E, National Instruments, Austin, TX, USA). The sound signal was first amplified (K1803, universal mono preamplifier, Velleman) before being acquired at 20 kHz. The forces and the position of the finger were acquired at a sample frequency of, respectively, 1 kHz and 500 Hz. A trigger signal allowed synchronization of the finger position with the force and sound signals.

### Data processing

The processing was done using a custom made Matlab software. The forces and positions were first low-pass filtered with a fourth-order, zero phase-lag Butterworth filter with a cutoff frequency at 30 Hz. This frequency was chosen to remove high frequency noise but permitted to keep a high temporal resolution. The sound was high-pass filtered (fourth order, zero-lag Butterworth) with a cutoff frequency of 25 Hz in order to remove DC component and low-frequency physiological artifacts (heartbeat, muscles contractions). The speed and acceleration along the scanning direction were derived from the position of the finger.

From each block of trials, we retained the three strokes that best complied with the following three conditions. The NF applied had to be in the range of the targeted force ±30%. There was no stick and slip during the movement (a stick and slip was detected by the presence of high peaks in the acceleration profile). The speed had to be maintained approximately constant for a range of 12 mm. The mean NF and TF, the mean coefficient of dynamic friction, μ = TF/NF, and the mean scanning velocity were then computed for each stroke. Due to the variability of the velocity adopted by each subject, we then separated all strokes into two velocity categories. The first category was ranging from 30 to 120 mm/s and the second was ranging from 120 to 250 mm/s. An estimate of the vibrations power was obtained by computing the root mean square (RMS) of the sound magnitude for each stroke.

**Figure 3:**
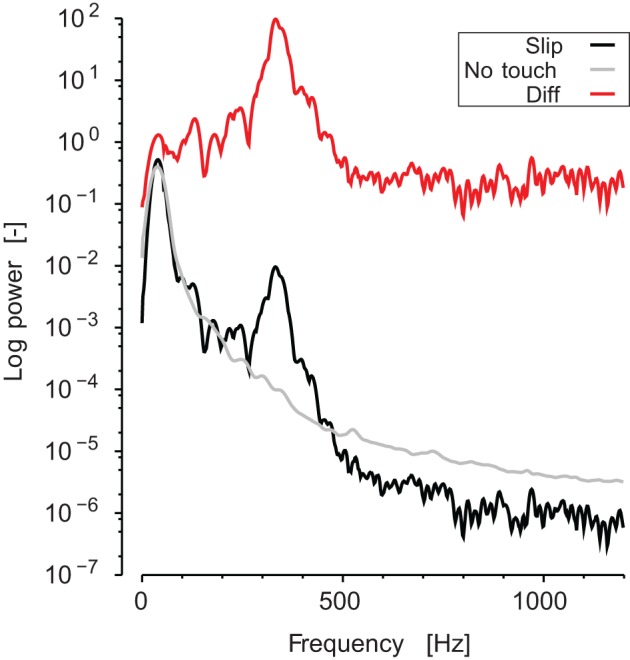
**Typical spectrum of the sound recorded**. Black line represents the averaged spectrogram during a slipping phase. Gray line represents the averaged spectrogram during a static no-touch phase. Red line is the ratio, which appears as a shift in log coordinates.

The analysis of the vibrations frequency content recorded at the wrist was conducted as follows. First, the short time Fourier transform (i.e., the spectrogram, Φ) of the sound was computed for each stroke. The width of the short-time window was 50 ms and the overlap was 45 ms (thus, a spectrum vector was computed every 5 ms). The frequency range was defined from 30 Hz to 1200 Hz. Using the same short-time windows, we computed the spectrogram of the sound during static and no contact periods for each block. This spectrum was averaged over time, and then subtracted from each stroke spectrogram in order to extract the signal due to the stroke itself for each trial (see Figure 3).

Second, we estimated the peak of intensity in each window of the spectrogram and extracted its corresponding frequency.

For periodic stimuli, the energy was expected to be precisely located at the mechanical fundamental frequency, *f*_0_ = *v*/λ_0_. The frequency of the peak in the spectrum was simply computed at each instant (*t*_i_) from,

(1) $$f_{p}\left(t_{i}\right)=\arg\underset{f}{\max}\left(\Phi \left(t_{i},f\right)\right).$$

The spatial period of the surface explored was estimated by computing the ratio between the scanning velocity and the frequency of the peak in the spectrum,

(2) $$\hat{\lambda }=\frac{v}{f_{p}}.$$

For non-periodic stimuli, no precise peak of intensity was expected. In order to get an estimation of the centroïd of energy in the spectrogram, we computed the median frequency of the spectrum,

(3) $$\text{such}\;\text{that}\sum_{f=30}^{f_{p}\left(t_{i}\right)}\Phi {\left(t_{i},f\right)}^{2}=\sum_{f=f_{p}\left(t_{i}\right)}^{1200}\Phi {\left(t_{i},f\right)}^{2},$$

and the estimate of the spatial periodicity of the surface was computed in the same way as for the periodic stimuli.

## Results

### Typical trials

**Figure 4:**
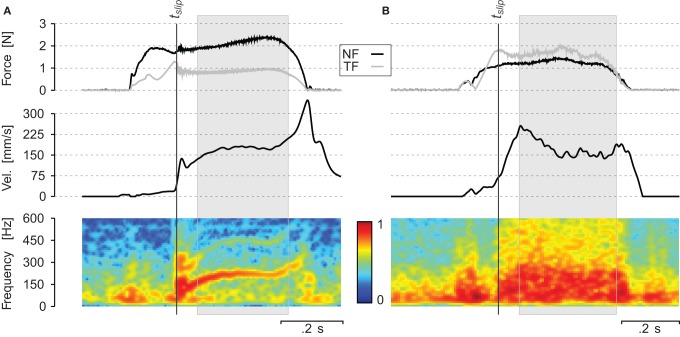
**Individual trial (A) on a periodic grating of spatial period λ = 0.8 mm and (B) on a sandpaper P240: evolution of normal force NF, tangential force TF, velocity of the finger *v* and spectrum of the sound over time**. *t*_*slip*_ corresponds to the onset of the slip. The gray box corresponds to the slipping phase. The spectrogram (lower part) was computed on 50 ms time windows and every 5 ms. Color represents logarithm of the power spectrum.

Two typical traces are shown in Figure 4. They illustrate the temporal evolution of the measured variables during the scanning of periodic grating **(A)** and sandpaper **(B)**. The target NF was 2 N for the periodic grating and 1 N for the sandpaper. The target scanning velocity was 150 mm/s for both. The upper panel shows the evolution of the NF (black line) and the TF (gray line). The central panel reports the finger scanning velocity. The lower panel shows the spectrogram of the sound recorded at the subject wrist. The subject touched the surface and adjusted the NF while progressively increasing the TF. Once the ratio TF/NF reached the static coefficient of friction, the finger started to slip at a nearly constant velocity. During the slip phase (gray box), vibrations propagated through the finger and hand tissues up to the wrist. For the periodic stimuli (see Figure 4A), the energy of the spectrum was concentrated around the fundamental frequency *f*_0_ = *v*/λ_0_ and there was energy in the harmonics also. For sandpaper (Figure 4B), the energy of the spectrum is spread over a very wide range during the whole slip. One can notice that at the beginning of the slip, there is a high peak of sound intensity, which is spread over a wide range of frequencies in both conditions.

### Spectral analysis

**Figure 5:**
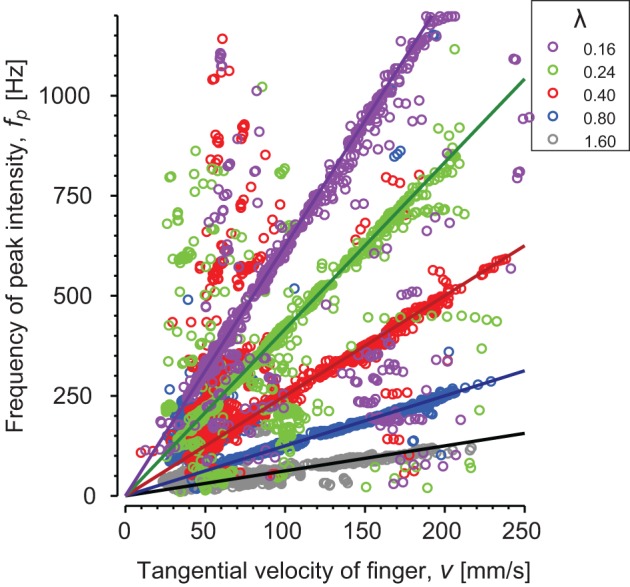
**Periodic stimuli: relationship between the speed of the finger and the frequency of the peak intensity of the spectrum for one individual subject**. Each stimulus spatial period λ) is represented by a different color: λ = 0.16 mm (magenta), λ = 0.24 mm (green), λ = 0.4 mm (red), λ = 0.8 mm (blue) and λ = 1.6 mm (gray). Continuous lines are theoretical slopes ($\text{f}=\frac{1}{\lambda }\cdot \text{v}$).

The peak intensity frequencies (*f*_*p*_) are plotted as a function of velocity for subject S3 (Figure 5). Each color corresponds to a different periodic stimulus. The thick line represents theoretical fundamental, λ_0_ = *v*/*f*_0_, and data points are *f*_*p*_ values computed for each window in the short time Fourier transform. Most of the points are found around the fundamental frequency and *f*_*p*_ was found correlated with the spatial periodicity of the stimuli up to 1.2 kHz. These results confirm the presence of vibrations correlated with the spatial periodicity of the stimuli and the scanning velocity.

**Figure 6:**
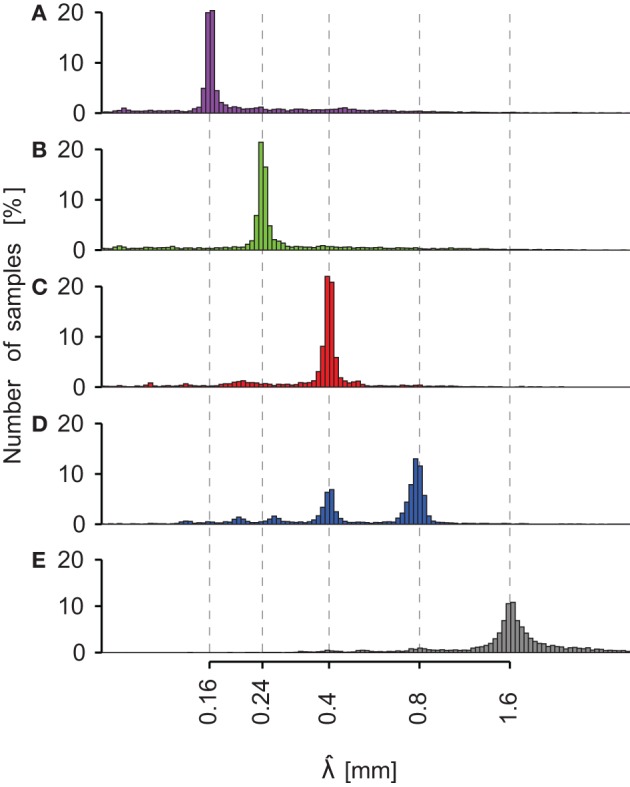
**Periodic stimuli: histogram of $\hat{\lambda }$ pooled for all subjects**. Each sub-axis corresponds to a different periodic stimulus. **(A)** λ = 0.16 mm (magenta). **(B)** λ = 0.24 mm (green). **(C)** λ = 0.4 mm (red). **(D)** λ = 0.8 mm (blue). **(E)** λ = 1.6 mm (gray). Dashed lines represent the actual spatial period of the stimuli. For each periodic grating, the principal mode corresponds to the theoretical value. The x-axis has a logarithmic scale. Logarithm bin width equals 0.015.

The histograms of spatial period estimates are plotted on Figure 6 for all trials (six subjects, three NF and two velocities) performed with the five different stimuli (0.16 mm < λ < 1.60 mm). The dashed lines are aligned on the actual spatial period of each stimulus. It can be clearly seen that the principal mode of the estimate of the spatial period for each surface is aligned on the actual spatial period. For some trials, $\hat{\lambda }$ was also found on the second, third or next harmonics (see Figures 6C and D). The principal modes observed for each stimulus and the numbers of observations are summarized in Table 4.

**Table 4:** **Principal modes for the estimate of spatial period $\hat{\lambda }$ for periodic and non-periodic stimuli**.

	**Periodic gratings**					**Sandpapers**		
	**P1**	**P2**	**P3**	**P4**	**P5**	**P800**	**P240**	**P80**
Principal mode (mm)	0.163	0.237	0.394	0.804	1.64	0.269	0.288	0.279
Percentage of observations (%)	57.49	59.78	68.02	49.42	59.73			

The percentage of observations corresponds to the fraction of the observations that have a spatial period within ±20% of the spatial period of the stimuli.

Spectral information related to sandpapers is summarized in Table 4. In contrast with the periodic stimuli, the identified spatial period varied much more within the same sandpaper grit size. Furthermore, there was no significant difference in the principal mode observed between the different grit sizes.

From the spectrum analysis, we could thus extract relevant information for the discrimination of periodic stimuli but not for sandpaper.

### Vibration magnitude

The RMS intensity of the sound was computed for each stroke. This parameter was computed as an indicator of the vibration magnitude transmitted up to the wrist. Assuming that the cavity pressure is uniform (the wave length of the vibrations is more than 30 cm at 1 kHz), the skin average displacement over the surface of the belt aperture was of the order of 0.3 μm (see Table 5). For periodic stimuli, a two ways ANOVA (no interaction term) revealed a significant effect of NF (*p* < 0.001) but not of velocity on the vibrations magnitude. The larger the force, the more intense was the vibration. A linear regression revealed a significant slope of 0.13 of the logarithm of intensity as a function of the NF. For sandpapers, we found no effect of velocity.

**Table 5:** **Normalization factors for pressure variation and skin displacement for each subject (corresponding to 0 in the log scale of Figure 7)**.

	**S1**	**S2**	**S3**	**S4**	**S5**	**S6**
**GRATTINGS**						
Pressure variation (Pa)	2.52	2.78	0.47	2.00	1.63	1.12
Skin displacement (μm)	0.35	0.39	0.06	0.28	0.23	0.16
**SANDPAPER**						
Pressure variation (Pa)	3.18	2.37	3.08	1.99	2.06	2.38
Skin displacement (μm)	0.44	0.33	0.43	0.28	0.29	0.33

The skin displacement was computed according to ∂*V* = ‒β*V*∂*p*, with β being the air compressibility, V the belt volume and p the pressure. We considered the stethoscope belt as a cylinder, and the skin acting like a piston.

**Figure 7:**
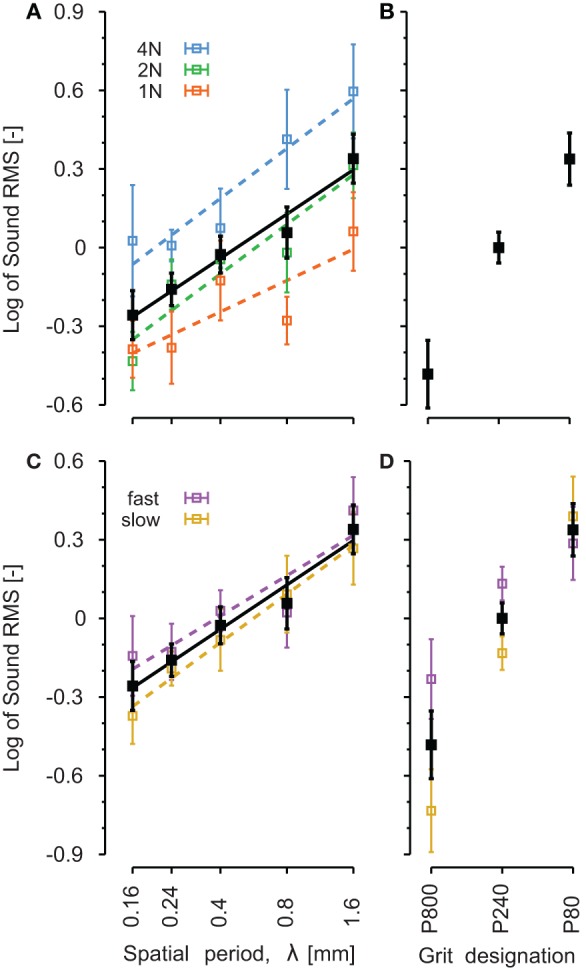
**Comparison of root mean square of the sound intensity on the different explored surfaces**. Black traces correspond to all conditions pooled together and are identical for **A,B** and **C,D**. Colored traces correspond to different level of normal forces **(A)** and different finger velocities **(C,D)**. Periodic gratings **(A–C)**: λ is the spatial period of the periodic stimulus. The x-axis has a logarithmic scale. Sandpapers **(B–D)**: from smooth (P800) on the left to rough (P80) on the right. The lines represent linear regressions on means. Error bars indicate the standard error of the mean.

The logarithm of the intensity is plotted on Figure 7 as a function of the five stimuli's spatial period **(A–C)** and the three sandpaper's grit size **(B–D)**. The range of RMS values varied a lot across subjects. Before pooling the data of all subjects, the data of each individual were normalized with respect to a specific condition: λ = 0.4, NF = 2 N and *v* = 100 mm/s for periodic stimuli and P240, NF = 1 N and *v* = 100 mm/s for sandpapers. Normalization factors are shown in Table 5 for each subject.

Figure 7 presents data for all speeds and forces pooled together in black lines. The effect of NF (Figures 7A,B) and finger velocity (Figures 7C,D) is shown in colored lines. For periodic stimuli, the logarithm of the RMS magnitude increased linearly with the logarithm of the spatial period (slope = 0.24, *p* < 0.001, all forces pooled). The slope was significant for each level of force separately (*p* = 0.029, *p* < 0.001, and *p* = 0.003 for NF equal to 1, 2, and 4 N, respectively). There was a significant pair wise difference between each of P1-P2-P3 and P5 (*p* < 0.05, Tukey's test) and between P1 and P4 (*p* < 0.05, Tukey's test).

For sandpapers (Figure 7B), the grit size induced a significant increase of the vibration RMS (*p* < 0.05).

### Coefficient of friction

**Figure 8:**
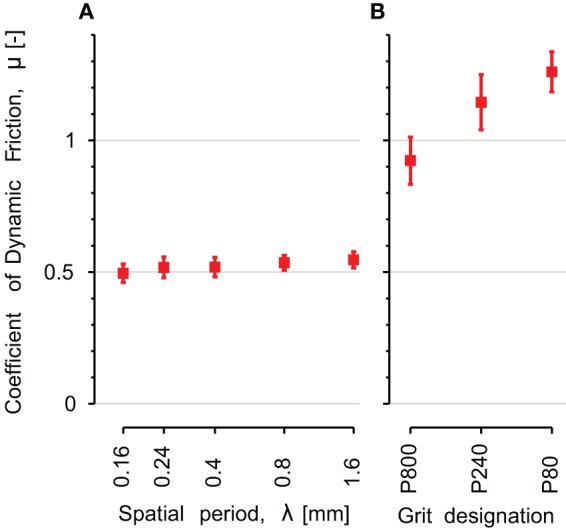
**Comparison of coefficient of dynamic friction on the different explored surfaces. (A)** Periodic gratings: *λ* is the spatial period of the periodic stimulus. The x-axis has a logarithmic scale. **(B)** Sandpapers: from smooth (P800) on the left to rough (P80) on the right. Error bars indicate the standard error of the mean.

The coefficient of dynamic friction is plotted on Figure 8 as a function of the five stimuli's spatial period **(A)** and the three sandpaper's grit size **(B)**. For the periodic stimuli the coefficient of friction was small (around 0.5) and constant across the different stimuli (*p* = 0.86).

The dynamic friction was much higher for the sandpapers, ranging from 0.9 for P800 to 1.3 for P80. We found a significant pair wise difference between P80 and P800 (*p* = 0.04, Tukey's test).

## Discussion

Libouton et al. (2012) suggested that the somatosensory system was able to collect roughness from other sources of information than fingertip afferences. The authors highlighted that the tactile roughness discrimination performance was unaffected by entrapment or traumatic section of the median nerve at the wrist. As a consequence, they concluded that, if the innervation of the finger pad was compromised, information about textures could be captured and encoded by remote mechanoreceptors located in more proximal tissues where the innervation was intact. We attempted to explain the mechanism through which humans can detect textured surfaces and discriminate between them when the innervation of the finger pad is radically compromised.

To this end, we recorded vibrations in the range from 30 Hz upto 1.2 kHz taking place in subjects' wrist tissues while they explored textured surfaces. Our results demonstrate the propagation of vibratory waves produced by the finger pad interaction with a textured surface to at least the wrist regions. We found that the vibration spectral energy was located on the fundamental frequency plus harmonics for periodic stimuli. This frequency, together with the fingertip velocity, corresponded to the spatial period of the stimulus. We did not find any obvious features that could reflect the grit size in the spectra for sandpapers. We also found that the coefficient of dynamic friction varied according to the sandpapers' grit sizes but remained constant across the different polyurethane gratings. These results corroborate the findings of Wiertlewski et al. (2011b) who measured the vibrations taking place at the finger-surface interface and found that the transformation of the surface geometry into vibrations was strongly non-linear, causing both frequency spread and background noise.

The present results show that the biomechanics of hand tissues can transmit surface interaction signals far away from the contact, up to the forearm, and probably much beyond. The likely mechanical paths are bone conduction (Corso, 1963), tendon conduction (Pourcelot et al., 2005), surface Rayleigh waves (Liang and Boppart, 2010), shear waves in the bulk (von Gierke et al., 1951); or a combination thereof. Our results establish the possibility for the somatosensory system to combine information from multiple modalities and from distributed locations in order to assess properties of the characteristics of an unknown texture, using the fingertips as a distant probe and through a variety of different mechanisms yet to be described.

When rubbing a finger against sand paper, the spatial periodicity of the vibration signal in the wrist was in the range of the fingerprint periodicity, i.e., near 0.3 mm (see Table 4), suggesting that the fingerprints could make a spectral selection due to their intrinsic periodicity (Martinot, 2006; Prevost et al., 2009; Scheibert et al., 2009). For the periodic stimuli, some estimates of the spatial period were around 0.4 mm, especially for 0.8 mm gratings (see Figure 4D), which could also be linked to the fingerprint periodicity although a more likely explanation is simply a frequency doubling effect in a preferred range due to the presence of two edges per period of the stimulus.

It was hypothesized that the Pacinian channel could encode vibratory intensity (Hollins and Bensmaïa, 2007). This population of receptors is indeed a good candidate to encode scanned textures through remotely transmitted signals. Moreover, the exquisite sensitivity of the Pacinian corpuscule makes them good candidates for picking up signals having travelled over considerable distances (Hunt, 1961), see Hamann (1995) for a review. Significant vibrations in the wrist imply that, in healthy subjects, very large populations of receptors can be stimulated throughout the hand and the arm.

In the present study we have demonstrated that vibrations generated during the scanning of textured surfaces propagate through the finger and hand and stimulate receptor populations in regions far away from the contact region at least up to the wrist. The spectrum and the magnitude of the measured vibrations have to be considered as the best predictors of roughness discrimination. These findings have important implications in providing realistic sensory feedback for prosthetic-hand users. Indeed, it suggests that advanced prosthetic arms, equipped with sensory feedback, could partially restore the tactile sensation of amputees, through the activation of the remote mechanoreceptive system. Therefore, the transmission and reception of vibratory stimuli related to texture to the remaining stump becomes a major priority in the conception of future prosthetic hands.

## Conclusion

We showed the presence of high frequency vibrations in the wrist of subjects exploring rough surfaces. The vibratory power is the best predictor of the texture roughness sensed remotely. It accounts for both periodic and non-periodic surfaces, with very different frictional properties. This result is consistent with perceptual studies carried on either with fingertip touch or with probe scanning. Moreover, we showed that the frequency content of periodic stimuli contained also potential information to encode roughness. Thus, many questions remain unclear about the contribution of the spectral content of the vibrations in the perceived roughness. Finally, friction may contribute to refine perception of roughness.

### Conflict of interest statement

The authors declare that the research was conducted in the absence of any commercial or financial relationships that could be construed as a potential conflict of interest.
